# Role of Metformin in the Management of Polycystic Ovarian Syndrome-Associated Acne: A Systematic Review

**DOI:** 10.7759/cureus.28462

**Published:** 2022-08-27

**Authors:** Humaira Shamim, Marie Jean, Muaaz Umair, Pratyusha Muddaloor, Michelle Farinango, Akhil Ansary, Amulya Dakka, Zahra Nazir, Chantelle T White, Ahmad B Habbal, Lubna Mohammed

**Affiliations:** 1 Dermatology, California Institute of Behavioral Neurosciences & Psychology, Fairfield, USA; 2 Dermatology, College of Physicians and Surgeons Pakistan, Lahore, PAK; 3 Psychiatry, California Institute of Behavioral Neurosciences & Psychology, Fairfield, USA; 4 Internal Medicine, California Institute of Behavioral Neurosciences & Psychology, Fairfield, USA; 5 Research, California Institute of Behavioral Neurosciences & Psychology, Fairfield, USA; 6 Clinical Sciences, St. Martinus University Faculty of Medicine, Willemstad, CUW; 7 Internal Medicine Clinical Research, California Institute of Behavioral Neurosciences & Psychology, Fairfield, USA; 8 Psychology, California Institute of Behavioral Neurosciences & Psychology, Fairfield, USA; 9 Cardiology, California Institute of Behavioral Neurosciences & Psychology, Fairfield, USA

**Keywords:** polycystic ovarian syndrome, antiandrogen, hyperandrogenism, metformin, nodulocystic acne, acne

## Abstract

Metformin, a biguanide hypoglycemic agent that is safe and effective for treating acne in women with the polycystic ovarian syndrome (PCOS), has shown growing evidence of improving insulin resistance, hyperandrogenism, dyslipidemia, overall cardiovascular health, quality of life, psychological wellbeing, and general health outcomes. This study aims to identify and summarize the effects of metformin in patients with PCOS-associated acne. This systematic review was based on the Preferred Reporting Items for Systematic Reviews and Meta-Analyses (PRISMA) guidelines. A systematic search was done on PubMed, PubMed Central, Cochrane, Google Scholar, and Science Direct databases from 2011 up to 23 February 2022. Randomized controlled trials (RCTs), cross-sectional studies, observational studies, literature reviews, systematic reviews, and meta-analyses published in English were selected. The data was extracted to a predefined template. Each study was individually checked by using a quality assessment. The initial search generated a total of 218 studies. Nine studies were included in the final selection: two RCTs, one hospital-based longitudinal study, one hospital-based clinical trial, three cross-sectional studies, three systematic reviews with meta-analyses, and one narrative review. Metformin is generally effective and safe for improving PCOS-associated acne and the quality of life. More clinical trials are required to determine the indications for prescribing metformin in patients with PCOS-associated acne.

## Introduction and background

In 1935, Stain and Leventhal first described polycystic ovarian syndrome (PCOS) in a series of seven women who presented with bilateral cystic ovaries, amenorrhea, hirsutism, and obesity [1]. Rotterdam criteria have been used to diagnose PCOS over the last 10 years. 

PCOS affects a considerable proportion of women, particularly South Asians residing in the United Kingdom [2-4]. Fifty-two percent of women residing in the Indian subcontinent have polycystic ovaries, the highest reported prevalence [5]. PCOS is associated with interference in reproductive hormones like luteinizing hormone (LH), follicle-stimulating hormone (FSH), estrogen, and testosterone which result in menstrual cycle irregularities like oligomenorrhea or amenorrhea. Acne is the most common manifestation of hyperandrogenism among other cutaneous manifestations like acanthosis nigricans, androgenic alopecia, and hirsutism. Acne vulgaris, characterized by chronic inflammation of the pilosebaceous unit, is one of the most common skin diseases for routine dermatology outpatient visits among patients aged 15 to 40 years [6]. Hyperandrogenism should be considered in female patients with sudden onset of severe acne that is associated with hirsutism or irregular menstrual periods. It is a risk for reproductive complications like infertility or subfertility, metabolic complications, cardiovascular risks, and psychological disturbances, along with type 2 diabetes and endometrial dysplasia [7-8]. Metformin is a biguanide that is effective and safe for improving insulin resistance, menstrual cycle irregularities, acne, and symptoms of hyperandrogenism like hirsutism [9]. Metformin’s mechanism of action is still not clear. While it is known to be an insulin-sensitizing agent which acts by inhibiting gluconeogenesis and lipogenesis, thus enhancing glucose uptake in the liver, skeletal muscles, adipose tissue, and ovaries. Metformin also decreases ovarian androgen production by 20% to 25% [10].

The role of metformin in acne and PCOS has been mentioned in 11 RCTs and nine cohort studies. All are shown to describe the remarkable improvement of PCOS-associated acne, except for three studies [11-12]. PCOS has a genetic predisposition and other multifactorial causes which include neuroendocrine, lifestyle, environmental, and obesity.

Hyperandrogenism, insulin resistance, compensatory hyperinsulinemia, and an imbalanced ratio of LH to FSH produce a metabolic disturbance and affect the ovaries and endometrium [11]. The exact etiology is an enigma. 

The management of PCOS is a multidisciplinary approach that includes modification of general lifestyle by endocrinologists, gynecologists, dermatologists, and psychologist consults. Patients with PCOS are commonly seen first by a dermatologist [11]. The underlying hyperandrogenism that characterizes polycystic ovarian syndrome clinically manifests also as hirsutism and menstrual irregularities. Metformin also leads to clinical improvement in PCOS-associated acne. The pathophysiology of acne vulgaris is related to chronic inflammation of pilosebaceous follicles, increased sebum secretion, and colonization of Propionibacterium acnes [11]. This study aims to explore the role of metformin in PCOS-associated acne.

## Review

Materials and methods

This study is done using the Preferred Reporting Items for Systematic Reviews and Meta-Analysis (PRISMA) to design and describe the findings of this systematic review. Search strategy: A primary literature search was conducted using PubMed, Google Scholar, Cochrane, and Science Direct. Articles from the last 10 years on females with PCOS-associated acne aged 19 to 44 years treated with Metformin were selected if they also met the criteria, for example, full-text articles in English and conducted from 2011 up to 23 February 2022. Using the MeSH search function, the keywords “polycystic ovarian syndrome”, “metformin”, “hyperandrogenism”, and “acne” were used.

The PubMed was explored with Medical Subjects Headings [MeSH] controlled vocabulary or search items as follows "Polycystic Ovary Syndrome/diet therapy"[MeSH] OR "Polycystic Ovary Syndrome/drug therapy"[MeSH] OR "Polycystic Ovary Syndrome/metabolism"[MeSH] OR "Polycystic Ovary Syndrome/physiopathology"[MeSH] OR "Polycystic Ovary Syndrome/statistics and numerical data" [MeSH] OR "Polycystic Ovary Syndrome/therapy" [MeSH] OR PCOS AND ANDROGEN ANTAGONISTS"[ALL FIELDS] OR "ANDROGEN ANTAGONISTS"[MESH TERMS] OR ANTIANDROGENS [TEXT WORD] OR METFORMIN [TEXT WORD] OR HYPERANDROGENISM AND ACNE OR ["acne vulgaris"] [MeSH Terms] OR acne vulgaris [Text Word] OR ["polycystic ovary syndrome"] [MeSH Terms] OR NODULOCYSTIC ACNE OR polycystic ovarian syndrome [Text Word] We applied the Boolean method to combine the keywords and MeSH terms and synthesize a uniform through searching various databases. 

A total of 218 articles were included, which included 190 from PubMed, 25 from Google Scholar, two from the Cochrane database, and one from ScienceDirect. PubMed included 190 articles after MeSH searching - 166 records were irrelevant, and eight duplications were removed. Finally, 14 reports were evaluated for eligibility and quality.

The inclusion and exclusion criteria used in this review are presented in Table 1 below.

**Table 1:** Inclusion and exclusion criteria.

Inclusion criteria	Exclusion criteria
English language	The presence of any dermatological disease besides acne. The Presence of any systematic disease. Pregnancy and lactation. Females of non-reproductive age group. Acne due to other causes like the use of steroid hormones, antidepressants, or Alcohol.
Articles free full text	Animal studies. Non-English language articles Study Selection 218 articles from the last 10 years were taken into consideration, including traditional reviews, systematic reviews, meta-analyses, bibliography, books and documents, case reports, classical articles, clinical studies, clinical studies, and clinical trials.
Female patients of PCOS in reproductive (age group: 19-44 years)	Two researchers independently screened all full free text articles titles and abstracts to be included and extracted the relevant studies, animal studies, and articles not written in the English language were excluded. The two researchers resolved disagreements over eligibility by studying the study design, intervention implemented, outcome measured, and the relevance to our inclusion and exclusion criteria. The final decision on study selection was reached by discussion.

Study quality appraisal

We studied each article separately for the potential risk of bias. A comprehensive analysis of quality was conducted. The systematic reviews and meta-analyses were subjected to assessment using the multiple systematic reviews (AMSTAR) 2 tool [12]. The clinical trials and randomized control trials (RCT) were critically evaluated with the Cochrane Risk of Bias 2 (RoB2) tool. Each study was scrutinized based on seven criteria to find potential biases. The Newcastle Ottawa Scale (NOS) was used to assess the quality of cohort studies [13]. Each criterion was scored as either high quality, low quality, or unclear. Initially, 218 articles over the last 10 years were identified through search terms across four literature databases. 

The final 11 articles were short-listed, as mentioned in the PRISMA chart [14].

**Figure 1:**
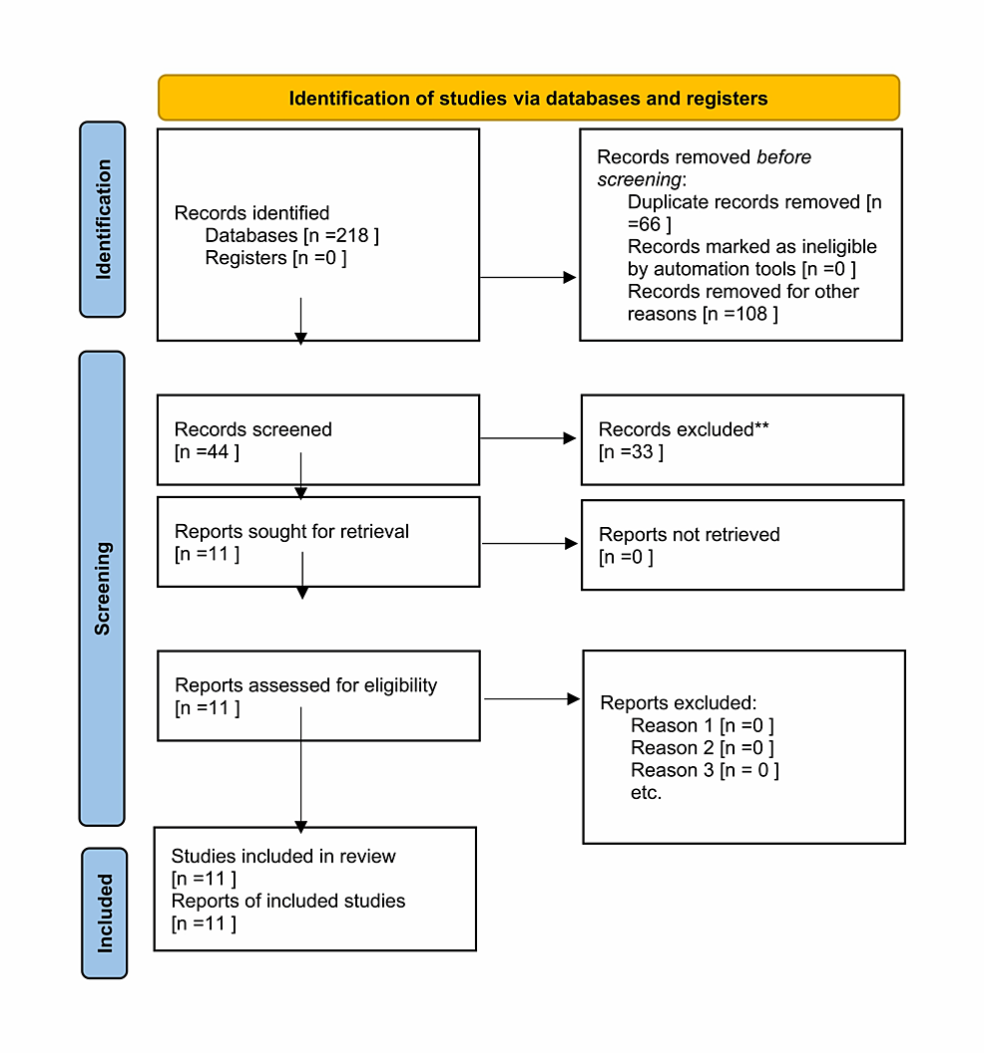
PRISMA chart

Figure 1 shows the PRISMA chart.

Discussion

Acne, hirsutism, androgenic alopecia, acanthosis nigricans, and seborrheic dermatitis are common cutaneous manifestations of PCOS. Insulin resistance plays a pivotal role in polycystic ovarian syndrome [15,16]. The biguanide metformin (N, N-dimethyl biguanide) is a hypoglycemic drug that improves insulin sensitivity, decreases insulin levels, and corrects ovarian and functional adrenal hyperandrogenism in PCOS. Metformin also leads to clinical improvement in PCOS-associated acne [17,18].

Dermatologists encounter acne vulgaris in patients of age groups ranging from 14 to 40 years of age [19-20]. Metformin decreases hepatic glucose output and increases glucose availability for muscles and adipose tissues by reducing insulin resistance. Other commonly used treatment modalities in acne vulgaris are oral tetracyclines, azithromycin, isotretinoin, and topical agents like benzoyl peroxide, adapalene, topical tretinoins, and miscellaneous other topical agents. In addition, hormonal imbalance plays a determining role in the clinical development of acne. Androgens increase sebum production by activating the activity of sebaceous glands. Colonization of Propionibacterium acne plays a pivotal role in the pathogenesis of acne formation, which may present as papules, pustules, nodules, cysts, and scarring [21].

Major sources of androgens 

Major sources of androgens are ovaries and adrenal glands. Ovaries produce androstenedione and testosterone. Adrenal glands produce dehydroepiandrosterone sulfate [DHEA], androstenedione, and testosterone [19]. Clinical assessment of acne is usually graded by using Acne Global Severity Scale.

The US FDA Acne Global Severity Scale categorizes acne according to the following five categories: 

"1) Clear, with no inflammatory signs. 2) Almost clear, with no more than one pustule or papule. 3) Mild, some non-inflammatory lesions with no more than a few papules or pustules but no evidence of any nodule. 4) Moderate, up to many non-inflammatory lesions, may have some inflammatory lesions but no more than one small nodule. 5) Severe, up to many non-inflammatory and inflammatory lesions, but no more than a few nodules."

Acne vulgaris is common in almost 80% of teenage females and may or may not be associated with PCOS. When acne vulgaris is associated with PCOS, the following changes in the serum may be seen.

Hormonal serum analysis 

Raised DHEAS, raised total free testosterone, raised androstenedione, reduced sex hormone binding globulin SHBG, raised LH, raised FSH, raised serum prolactin, raised insulin, high insulin-like growth factor binding protein IGFBP-1 and insulin-like growth factor IGF-1.

Psychological effects of PCOS

Patients with PCOS likely suffer from a wide range of psychological problems like low self-esteem, mood swings, depression, anxiety, and even psychosis. In PCOS, acne and hirsutism are the most common markers of affecting the quality of life by worsening the emotional well-being of women [21]. 

Cutaneous manifestations of PCOS 

The cutaneous features of PCOS are the first to develop clinically. Early diagnosis is possible by screening the suspected patients, and prompt treatment to reduce complications and improve quality of life. These cutaneous features, like acanthosis nigricans, are closely related to insulin resistance. The raised levels of insulin act through insulin-like growth factor receptors to cause acanthosis nigricans, a skin condition characterized by thick and dark velvety skin in the axilla, groins, neck, and frictional surfaces [20,21]. Hyperandrogenism has clinical cutaneous findings of acne, hirsutism, androgenic alopecia, and seborrhea.

Reproductive and neoplastic complications

Obesity, infertility, hirsutism, and acne negatively affect the quality of life of women with PCOS. This latter is characterized by amenorrhea or anovulation, which is associated with infertility. Among the other gynecological complications, miscarriage, endometrial hyperplasia, and endometrial carcinoma have been documented.

Raised LH levels are involved in the pathogenesis of PCOS as a result of increased pulse frequency of hypothalamic gonadotropic hormone (GnRH) or low levels of progesterone resulting from oligo-or anovulation [19,22]. These patients can develop subfertility or infertility. The altered FSH- to LH ratio stimulates the ovarian theca cells to produce androstenedione. High levels of insulin also play a key role in PCOS pathogenesis.

PCOS manifests as hyperinsulinemia and hyperandrogenism and raises the level of the luteinizing hormone LH [19,22]. This alters the normal pituitary ovarian axis, which leads to the clinical presentation of oligomenorrhea, infertility, obesity, hirsutism, acne, hypertension, and hyperlipidemia [22]. The use of metformin leads to significant improvement of hyperandrogenism and its clinical manifestations.

**Figure 2:**
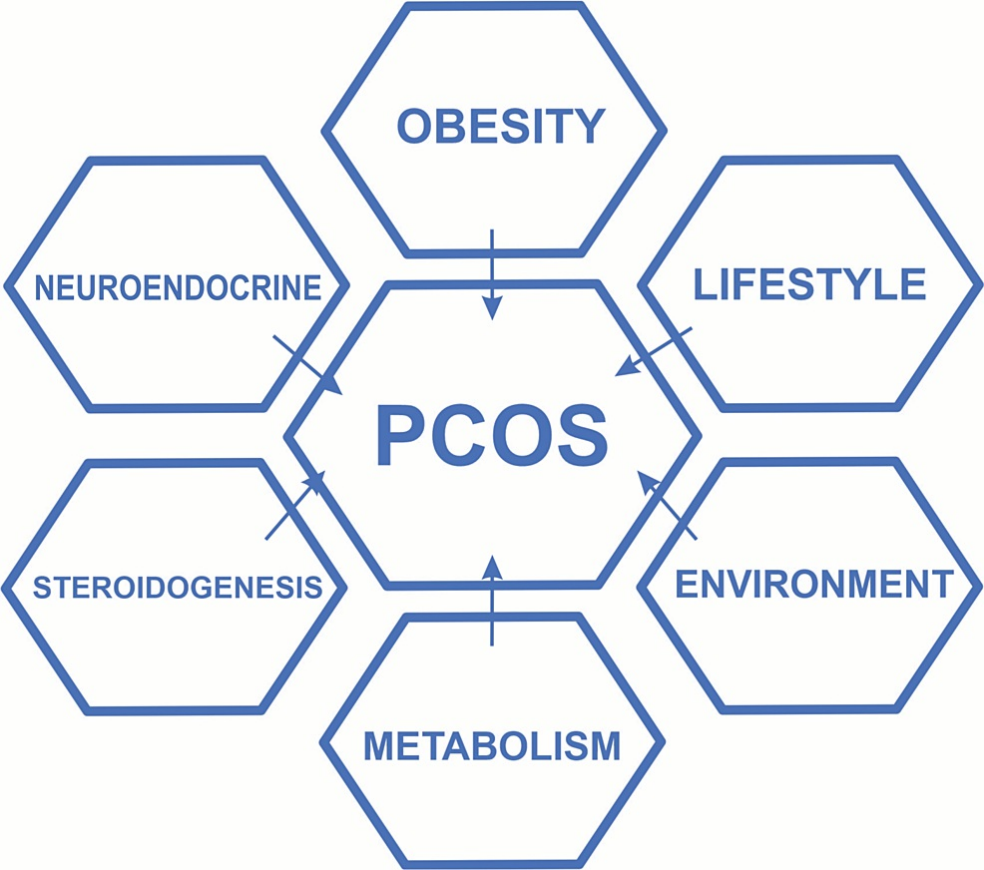
Etiology of PCOS. Figure modified by Shamim H.

 Figure 2 explains the etiology of PCOS [23].

Table 2 explains different diagnostic criteria of PCOS [23].

**Table 2:** Diagnostic criteria of PCOS. ESHRE/ASRM. European Society for Human Reproduction and Embryology/American Society for Reproductive Medicines; NIH/NICH National institutes of Health/National institute of Child Health and Human Disease

NIH/NICHD 1992	ESHRE/ASRM (Rotterdam Criteria) 2004	Androgen Excess Society 2006
Exclusion of other causes of androgen excess or related disorders includes all of the following:	Exclusion of other androgen excess or related disorder includes two of the following:	Exclusion of other androgen excess and related disorders include all of the following:
1) Clinical or biochemical Hyperandrogenism	1) Clinical or biochemical Hyperandrogenism	1) Clinical or biochemical Hyperandrogenism
2) Menstrual Dysfunction	2) Oligo-ovulation or anovulation	2) Ovarian dysfunction or polycystic ovaries

Cardiovascular complications of PCOS

Insulin is produced by the pancreas. It helps to maintain the blood sugar level within the normal range. Hyperinsulinemia is usually linked to type 2 diabetes mellitus. However, symptoms of hyperinsulinemia include sugar cravings, weight gain, anxiety, sweating, frequent hunger, and fatigue leading to hypoglycemia. Metformin helps in ameliorating the symptoms of hyperinsulinemia in patients with PCOS. Metformin is reported to decrease hepatic glucose output, thereby decreasing the insulin requirement [24]. Women with PCOS are prone to hypertension, coronary vascular disease, and ischemic vascular disease [24]. Along with other common comorbidities, young women with PCOS are at risk of early-onset acute coronary vascular disease and endothelial dysfunction [25].

**Figure 3:**
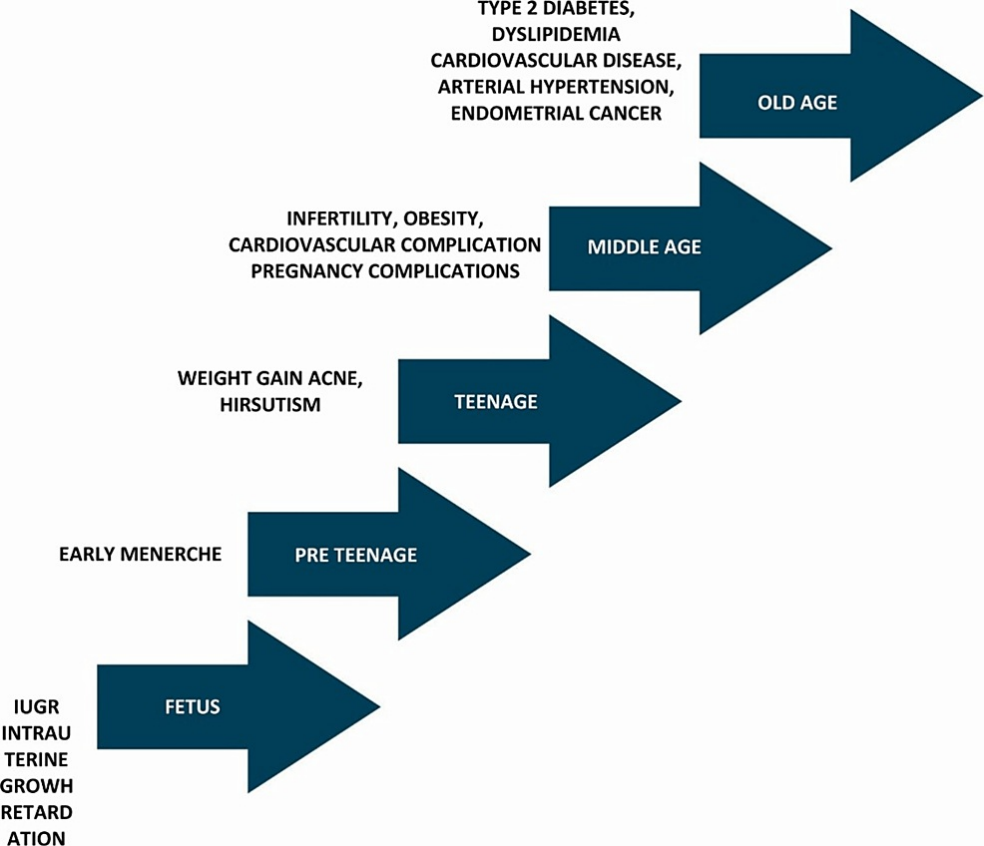
The effects of PCOS at different stages of life. Figure modified by Shamim H.

Figure 3 demonstrates the effects of PCOS at different stages of life [26].

Mechanism of action of metformin

The exact mechanism is unclear. Metformin is proposed to act by inhibiting mitochondrial function [27]. Creating an adenosine triphosphate-deficient environment causes an activation of adenosine monophosphate-activated protein kinase (AMPK) and upregulation of catabolic metabolism. Metformin diminishes the proinflammatory response by inhibiting nuclear factor kappa B (NF-kB) through both liver-related AMPK dependent and independent pathways, thus suppressing the production of nitric oxide (NO), prostaglandin E2 (PGE2), and acute inflammatory markers like tumor necrosis factor-alpha, interleukin-1, interleukin-6 [28].

Table 3 explains the study synthesis.

**Table 3:** Study synthesis table. DHEA: Dehydroandrosterone; PCOS: polycystic ovarian syndrome

Sr.	Author	Year of publication	Journal published	Type of study	Number of female patients with PCOS/number of articles	Purpose of study	Results	Conclusion
1	Timothy h. Schmidt et al. [17]	2016	JAMA Dermatology	Retrospective cross-sectional study 401 women with PCOS included	401 patients	To identify cutaneous findings in PCOS.	Hirsutism, acanthosis nigricans, and acne were associated with cutaneous findings in PCOS.	PCOS association with acne, hirsutism, and multiple other skin conditions.
2	John K Lee ms et al. [18]	2017	Dermatology Online Journal	A systematic review	Three clinical trials with 144 patients included.	To assess the role of metformin as an adjuvant for the treatment of acne vulgaris.	Reduction in acne after 12 weeks of metformin use.	Metformin may be an effective and safe adjunct treatment in the treatment of moderate to severe acne vulgaris.
3	G. Franik et al. [19]	2018	European Review for Medical and Pharmacological Sciences	Hospital-based clinical trial	110 patients with PCOS	To investigate the impact of hormonal and metabolic disorders of PCOS-associated acne vulgaris.	The severity of acne in PCOS is related to serum testosterone, DHEA dihydroando sterone	Acne global severity scale in PCOS women is associated with high total testosterone and dihydrotestosterone sulfate DHEA
4	M Artani et al. [20]	2018	Cureus Journal of Medical Science	Cross-sectional study 50 women PCOS included.		To determine the effects of metformin in polycystic ovarian syndrome.	Metformin improved acne, and menstrual irregularities, and hirsutism	Metformin has a significant role in PCOS women.
5	S Mukkamala et al. [21]	2018	Journal of Pakistan Association of the Dermatologist	A cross-sectional study	50 patients	To document various cutaneous manifestations in PCOS.	Acanthosis nigricans and acne are the most common cutaneous manifestation of PCOS.	Acanthosis nigricans to be the most common cutaneous finding in PCOS.
6	Shipli Sharma et al. [22]	2019	J Clin Aesthet Dermatol	Original research hospital-based interventional longitudinal study.	40 patients	To evaluate the efficacy of metformin therapy in women with PCOS-associated acne in terms of acne load.	Using the disease severity index DSI acne severity was reduced with metformin.	Suggested efficacy in PCOS.
7	Calvin t. Sung et al. [23]	2020	Journal of Drugs in Dermatology	A systematic review	26 articles were shortlisted	To assess evidence related to the use of metformin for treating primary cutaneous disorders.	Metformin is safe and efficacious in treating various cutaneous disorders, including acne and PCOS.	Metformin is safe and efficacious in PCOS and multiple other dermatological conditions.
8	Donna Vine et al. [24]	2021	Journal of the Endocrine Society	Randomized control trial	38 patients	To determine the effect of 12 weeks of high dose fish oil and metformin with metabolic syndrome and PCOS.	Fish oil and metformin significantly lowered fasting plasma triglycerides.	High fish oil and fish oil, metformin therapy tends to lower fasting and postprandial triglyceride levels.
9	J Bulsara et al. [25]	2021	Elsevier Journal endocrine and Metabolic Science.	Literature review article				More studies required for PCOS and lifestyle modification can improve quality of life in PCOS.
10	A Bahadur et al. [26]	2021	Cureus Journal of Medical Science	Randomized controlled trial	72 patients	To compare the efficacy of metformin alone and metformin combined with myoinositol plus d chiro-inositol in women with polycystic ovary syndrome.	72 patients were randomly allocated into two groups combined treatment has better results than metformin alone in these patients.	Combined therapy may have better results.
11	Jane l et al. [27]	2021	Diabetes ther 2021	A systematic review and meta-analysis				Equal efficacy of longer-acting versus metformin formulation but long-acting formulations being superior

Tolerability of metformin and potential adverse effects

Gastrointestinal disturbance is the most common adverse effect that limits patient compliance. Lactic acidosis is rarely seen. Metformin is frequently prescribed by endocrinologists. Metformin helps patients with PCOS in weight reduction. It also helps with metabolic comorbidities like dyslipidemia. Metformin acts centrally via suppressing hypothalamic neurons potentially and growth differentiation factor GDF-15 by reducing neuropeptide Y or by reducing appetite stimulation. There is also the involvement of gut-based mechanisms that increase the secretion of glucagon-like peptide GLP-1. Metformin improves lipid profile by inhibiting the activity of lipid synthesis [29].

The dose of metformin ranges from 750 to 2000mg. Metformin also helps the induction of ovulation in patients with PCOS. The side effects of metformin include nausea, diarrhea, flatulence, abdominal pain, and bloating. The small intestine has a concentration of metformin 30 to 300 times notably higher than plasma concentration [30]. The intestinal collection can be the reason for gastrointestinal side effects.

Limitations 

This review was limited by the types of papers found in the PubMed, Google Scholar, Cochrane, and Science Direct databases. Only papers in English from 2011 to 2021, and papers from Grey literature were excluded. The small number of articles directly studying the drug metformin in association with PCOS and small sample sizes.

## Conclusions

Metformin can have promising results in patients with PCOS. It may also potentially have positive effects on the quality of life. Further clinical trials are necessary. This summary of metformin in acne patients with PCOS recommends adequate efficacy, a high index of safety, and good tolerance. Thus, metformin can have a beneficial role in PCOS-associated acne.
